# Parent-Child Physical Activity Association in Families with 4- to 16-Year-Old Children

**DOI:** 10.3390/ijerph17114015

**Published:** 2020-06-05

**Authors:** Dagmar Sigmundová, Erik Sigmund, Petr Badura, Tomáš Hollein

**Affiliations:** Faculty of Physical Culture, Palacký University Olomouc, 77147 Olomouc, Czech Republic; erik.sigmund@upol.cz (E.S.); petr.badura@upol.cz (P.B.); tomas.hollein@upol.cz (T.H.)

**Keywords:** family, step count, dyads, children, adolescents, physical activity

## Abstract

Background: The main aim of this study was to quantify the associations between parents’ and children’s physical activity by age, gender, and the day of the week on the basis of a pedometer-measured step count (SC). Methods: The sample comprised data from 4-to 16-year-old children and their parents from the Czech Republic (1102 mother-child dyads and 693 father-child dyads). The parents and their children wore the Yamax SW200 pedometer during seven days of monitoring. Results: The strongest SC association was found between mothers and daughters aged 4–7.9 years on weekdays (r_p_ = 0.402; *p* < 0.01) and at weekends (r_p_ = 0.577; *p* < 0.01). In children aged 8–16, the parent-child association is gender-specific, with the father-son relationship being dominant, especially at weekends (weekend SC: fathers-sons_8–11.9 y_ r_p_ = 0.416, *p* < 0.01; fathers-sons_12–16 y_ r_p_ = 0.443, *p* < 0.01). An increase of 1000 steps in the fathers (mothers) is associated with an increase of more than 400 (200) steps in their sons (daughters). Conclusions: This study confirms a strong parent-child SC relationship in children younger than eight years of age. In older children, the parent-child SC association is gender-specific and dominated by the father-son relationship, particularly on weekends. The SC associations that are revealed can be used for the development of physical activity programs for adolescents.

## 1. Introduction

Physical activity (PA) is one of the main components of a healthy lifestyle [1]. In the Czech Republic, about one-third of adults have a physically inactive lifestyle [2,3]. Globally, an insufficient level of PA was found in 23.4% of adults in Central and Eastern Europe and 36.8% in high-income Western countries [4]. The rates of meeting the PA recommendations are even less favorable for school-aged children and adolescents. Only 35% of Czech children and youth (aged 6–17 years) achieve the recommended objectively monitored amount of PA as reported by Global Matrix 3.0 [5]. As expected, Czech preschool children achieve slightly better results in fulfilling the recommendations for PA (39–55%) [6] than older children. The results of the Health Behaviour in School-aged Children (HBSC) study suggest that only 22% of boys and 15% of girls aged 11–15 years met the guidelines for PA in the Czech Republic in 2018, which is line with the findings that the majority of adolescents do not meet current PA recommendations [7,8,9]. A high proportion of insufficiently physically active adolescents aged 11–17 years was also confirmed in other countries in Central and Eastern Europe [8,10]. This reported insufficient level of PA in young people, which has been observed globally, leads to a search for strategies and factors influencing PA levels in children and adolescents. These factors can include, for example, the environmental correlates of PA [11], the influence of socioeconomic status on PA [12], parent-child relationships in PA [13], or parenting style and parental support in relation to PA [14,15,16].

In line with self-determination theory [17], the level of children’s PA is closely related to their intrinsic motivation. In addition, self-determination theory states that parents, friends, and teachers are relevant for adolescents’ autonomous motivation to engage in PA [18,19,20]. Other theoretical concepts, including the social cognitive theory [21], social learning theory [22], ecological model [23], and human capital model [24] assume that parents and the family environment play an important role in promoting health-enhancing PA in children and adolescent [23,25]. The human capital model [24] describes parents as the basic and the first social influencer for sports and PA, with their main role in promoting PA being embedded in five socialization variables: initiation, encouragement, involvement, facilitation, and role modeling.

PA correlates vary with children’s age. While in younger children parental PA and parental support can be included among the PA correlates, in adolescents, positive associations were found between parents’ education, family or peer support, and PA [15,26,27,28]. Even though parental modeling in children [29,30] and adolescents [30] plays an important role in the adoption of healthy and physically active lifestyle behavior, parental support is also linked to children’s and adolescents’ PA, although the results of previous studies showed some inconsistencies in the associations observed [14,30,31]. Other correlates that play a role in parent-child PA association include children’s age [30], but also the gender of both children and parents. In parent-child studies, a dyadic approach is applied, i.e., the variables are measured in two members of the dyad (a child and a parent) [32]. Stronger associations in parent-child PA are expectable for mother-child dyads compared with father-child dyads, and between children and parents of the same gender, i.e., mother-daughter or father-son dyads [30,33,34,35].

There is a lack of studies using the dyadic approach to explain the relationship between parents’ and children’s variables on the one hand and physical activity on the other [36]. The strength of the dyadic approach, including monitoring of the PA of both parents and children by objective tools, lies in studying the mutual PA relationships and the possibility of explaining children’s PA in terms of parental modeling and involvement [13,30,36,37]. For these reasons, we decided to perform objective monitoring of the weekly PA (between 2013 and 2019) of both parents and their children with respect to their gender and the children’s age.

The main aim of this study is to quantify the associations between parents’ and children’s physical activity by age, the gender of both parents and their children, and the day of the week on the basis of a pedometer-measured step count (SC) and a dyadic approach.

## 2. Materials and Methods

### 2.1. Ethics

The study was approved by the Ethics Committee of the Faculty of Physical Culture of Palacký University Olomouc under No. 50/2012 (for families with school-aged children) and No. 57/2014 (for families with pre-school children). Participation in the research was voluntary, without any incentives.

### 2.2. Participants

A total of 3540 children aged 4–16 years and their parents from the Czech Republic (296 families with preschool children, 1610 families with children in the first to fifth grades, and 1634 families with children in the sixth to ninth grades of primary schools) were addressed. The participants were recruited from nine out of fourteen administrative regions, three of each in the lowest, middle, and highest thirds for gross domestic product per capita in the Czech Republic. In the second stage of sampling, the kindergartens and state primary schools were selected with respect to the distribution of the urban vs. rural population in the Czech Republic [38]. The parents were addressed through the schools (10 kindergartens and 51 primary schools) that their children attended. Before the monitoring was initiated, information meetings were held to describe the process/course of the research the children, parents, and teachers. Written consent to participate in the study was obtained from 2312 families (a response rate of 65.3%).

Research data was received from 4108 family members and other participants (1228 mothers; 777 fathers; 1015 daughters; 992 sons; and also *n* = 96 distant relatives, grandmothers, teachers, etc., who were excluded from the study). Family members (*n* = 605) who did not form a parent-child dyad were excluded from the study. Of the total number of parent-child dyads (*n* = 1990), 195 dyads were excluded because of non-compliance with any of the following criteria: children < 4 years old or ≥ 16 years old, pedometer wearing time < 8 h/day, missing data about body height or weight, disease during the monitoring week or step count data that covered less than four working days and one weekend day with a minimum daily step count of 1000 and a maximum daily step count of 30,000 [39]. The mean wearing days (excluding the first day) were 6.8 ± 0.68 days and mean wearing daily time was 819 ± 61 min. The final dataset included 1102 mother-child dyads and 693 father-child dyads. The basic characteristics of the participants are presented in Table 1.

### 2.3. Procedure

At the beginning, the parents were asked to record the anthropometric parameters of all the family members participating in the study (birth date, gender, body weight/height with 0.5-cm/kg accuracy). The parents were instructed how to measure their own body height and weight at home, as well as the height and weight of their children. The parental home measurement of the body height and weight of their children [40,41] is considered to be sufficiently valid for calculating body mass index (BMI) for the subsequent identification of excessive body weight in children [40].

On the basis of information obtained from the written consents received, pedometers and record sheets were prepared for the families. On the agreed date, a researcher came to the school to present information about the research and its purpose to the participants. Then the researcher distributed the pedometers and record sheets and instructed the participants how to handle the pedometer and how to write down the information on their PA on the record sheets. PA monitoring by means of pedometers began in the morning of the next day (after the meeting) for all the children and their parents. All participated children and adolescents regularly attended school during PA monitoring week. Both the children and their parents were instructed to wear the Yamax SW200 pedometer (Yamax Corporation, Tokyo, Japan) on their right hip all day except while sleeping and during water activities (swimming, bathing), as the pedometer was not waterproof. In addition, the pedometers were attached to the hip by a loop with a clip fastened to a pocket or belt. The participants wore the pedometer for eight consecutive days (the parents and their children on the same days), but the first day was a ‘trial day’ and was not included in the analysis because of possible reactivity [42,43]. On the ninth day, the participants returned the pedometer and weekly record sheet to a teacher who had received training. Each morning, the parents and their school-aged children recorded the time of attachment and step count; each evening they recorded the time of removal and step count. In the case of preschool children, this data was proxy-reported by their parents. The information from the weekly record sheet was re-written into the database and then exported to MS Excel for further processing. All the participants who completed seven days of PA pedometer monitoring received feedback on their PA level. The monitoring of weekly physical behavior was conducted regularly in the spring (58% of the families in the total sample) and autumn (42% of the families in the total sample) between 2013 and 2019. The data collection took place in several waves. In 2013, the study started with measuring in families with children aged 9–10 years; in 2014–2016, families with children aged 4–10 years were included in the study; in 2017, the study was expanded by the inclusion of families with children aged 11 years, and the families with adolescents (aged 12–16 years) were measured during 2018 and 2019.

### 2.4. Statistics

Statistical analyses were conducted using the IBM SPSS software, v. 22 (IBM Corp. Released 2013. Armonk, NY, USA). There were no indications for clustering by school; therefore, the results were analyzed in aggregate for family dyads. The differences in step counts by gender and by age groups were tested by means of the Mann–Whitney U test and Kruskal–Wallis test, respectively. The SC association between the parents and their children was examined by bivariate Pearson’s correlation. To quantify the increase in SC, linear regression was used with a dependent variable represented by the SC of the children (sons, daughters) and an independent variable represented by the SC of the parents (fathers, mothers). The alpha level of significance was set at a minimum value of 0.05.

## 3. Results

### 3.1. Daily SC of Family Members

**Figure 1:**
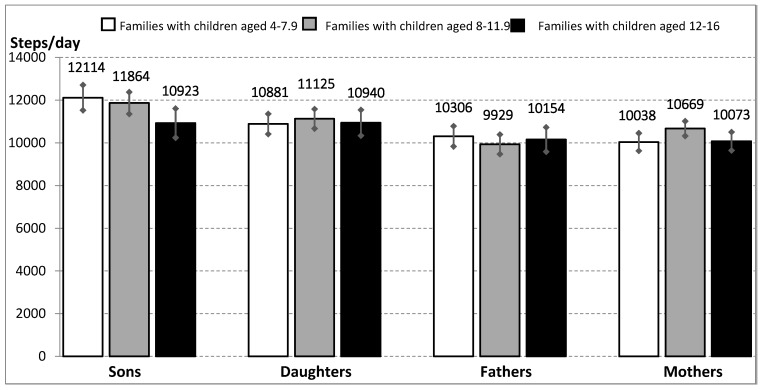
Daily step count (SC) of family members by children’s age category.

Generally, both boys and girls achieved the lowest SC on Sundays (10,249 ± 5749 and 9645 ± 5208, respectively); on the other hand, the highest SC was recorded on Fridays (12,219 ± 5806 and 11,549 ± 5229, respectively). Similarly, the mothers/fathers recorded the highest SC on Fridays (11,056 ± 5424/10,440 ± 5060) and the lowest SC on Sundays (9179 ± 5353/9390 ± 5031). The median values of daily steps over the seven-day monitoring period were 11,448 steps in the boys, 10,724 steps in the girls, 10,330 steps in the mothers, and 9526 steps in the fathers. Overall, in terms of weekly values, the boys showed significantly higher SC than the girls (*p* = 0.005); on the other hand, there were no significant differences in weekly SC according to the children’s age categories–in children (*p* = 0.053), mothers (*p* = 0.062), or fathers (*p* = 0.362). The weekly SC values of family members in relation to the children’s age category are presented in Figure 1.

### 3.2. Parent-Child Step Count Associations

The strongest SC association between parents and children (Table 2) was observed between mothers and daughters aged 4–7.9 years, especially at weekends (r_p_ = 0.577). In the youngest age category, the mother-child SC association was stronger compared with the father-child association, except the father-son association at weekends. Moreover, the strength of the mother-child associations in PA decreased with the children’s age, but father-child associations were relatively stable (especially in father-son relationships). In all age groups, mutual family relationships suggested that the mother-daughter associations were stronger than the mother-son associations and that the father-son associations were stronger than the father-daughter associations. This held true on working days, at weekends, and also in terms of all-week values.

**Figure 2:**
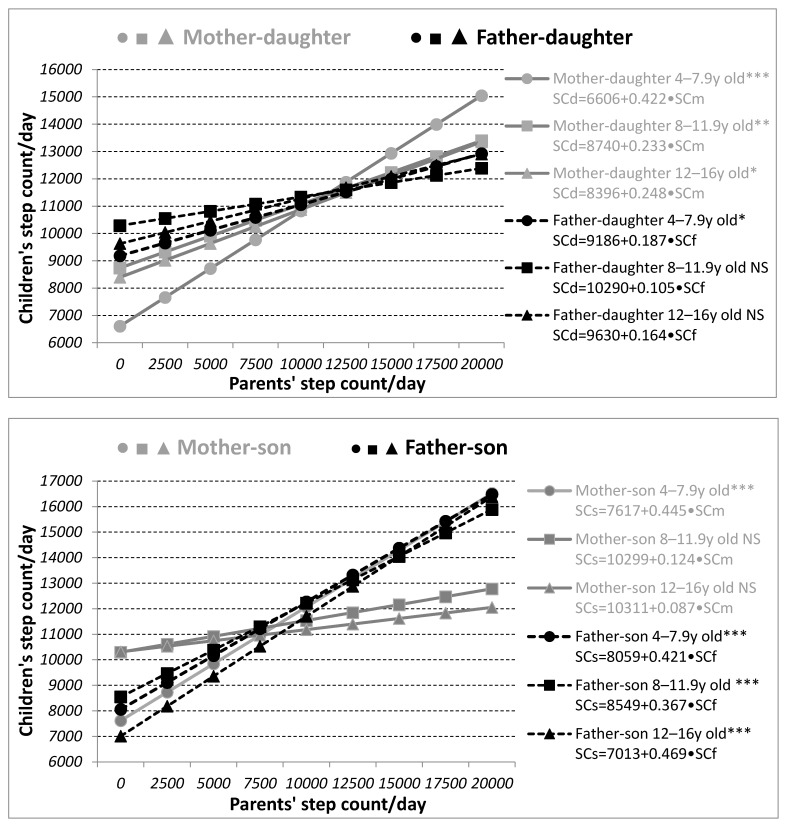
Relationship between parents’ and children’s weekly step count (SC).

The closest SC association was observed between the daughters and mothers, with an increase in the mothers’ daily SC by 1000 corresponding with 422 ‘additional’ steps/day in the youngest category, only 233 ‘additional’ steps/day in the category aged 8–11.9 years, and 248 ‘additional’ steps/day in the oldest category. The mothers’ physical activity was also linked positively to the physical activity of their sons aged 4–7.9 years, where an increase in the mothers’ SC of 1000 steps per day was related to an ‘increment’ of 445 steps per day in their sons. The sons’ SC correlated significantly with their fathers’ SC, with an increase in their fathers’ SC by 1000 steps per day of an ‘extra’ 421, 367, and 469 steps in the sons aged 4–7.9, 8–11.9, and 12–16 years, respectively (Figure 2).

SCd/SCs—step count of daughters/sons, SCm/SCf—step count of mothers/fathers. Statistical significance is expressed as NS—non-significant, * *p* < 0.05, ** *p* < 0.01, *** *p* < 0.001.

This section may be divided by subheadings. It should provide a concise and precise description of the experimental results, their interpretation as well as the experimental conclusions that can be drawn.

## 4. Discussion

Parents play an important role in promoting a healthy and active lifestyle in their children [14,28,44,45]. They are the first initiators of their children’s participation in sports and physical activity [24]. This study examines the parent-child dyad association in objectively measured daily life PA using pedometers. The results of this study extend the knowledge in the area of parent-child relationships concerning weekend and weekday PA with respect to children, as well as parents, of different ages and of both genders.

Consistently with other studies [27,46,47], it was observed that the boys were more physically active than the girls. Although it is difficult to increase the PA of girls, especially during adolescence, it seems that enjoyment and social support for PA are important mediators of adolescent girls’ PA [48]. Moreover, the offer of preferred activities such as dance, aerobics, and specific ball games can be a way of promoting higher-intensity PA in girls [49]. The sport preferences of Czech children and adolescents do not differ from other Central European countries. From individual sports, adolescents (15–18 years old) prefer cycling, swimming, downhill skiing, aerobics, and skating [50,51]. The most popular team sports include football, volleyball, floorball, ice hockey, and basketball [50]. This selection of activities is similar among those aged 10–14, with swimming, dancing, and skating being popular with girls and swimming, specific ball games, and skating with boys [52]. Knowledge of the preferred activities of boys and girls in childhood and adolescence can play a key role in creating effective intervention programs.

On the other hand, in the present study, no differences were observed in SC by age, although most studies confirm that younger children are more physically active than older ones [7,53]. In the current study, there is little evidence concerning the relationship between the parents’ PA and their children’s age. Consistently with a study that focused on objective measurement of PA in the context of parent-child pairs [30], the present study did not confirm significant differences in SC between groups of parents classified by the children’s ages.

Although the present study did not suggest any differences in SC with regard to the children’s age, the associations differed by age; a significant mother-child SC association and father-child SC association were found in the daughters and sons aged 4–7.9 years. In contrast, the Groningen Expert Center for Kids with Obesity (GECKO) study, which was based on objectively measured PA in children aged 4–7 years and self-reported PA in parents [27], confirmed a significant PA relationship only between mothers and daughters (not sons). This was analogous to a Greek study on five-to-eight-year-old children that reported that fathers’ PA was mainly related to their sons’ PA [47]. In the current study, we noticed stronger parent-child associations in children younger than eight years, whereas the SOPHYA study reported the strongest parent-child relationship in children aged 10–12 years [30]. Although a certain inconsistency can be seen among the studies [27,30,31,47], our findings imply that the strength of parent-child PA associations varies depending on the children’s age. The stronger parent-child associations identified in the youngest age group are aligned with parental modeling roles, which are important for promoting children’s physically active lifestyle [29,30].

The present study also confirmed a gender-specific parent-child SC relationship, which is consistent with a Brazilian study on parent-child associations [33]. Some studies also reported different results concerning parent-child PA associations with respect to the children’s or parents’ gender [30,33,54]. On the other hand, regardless of the children’s age, the present study suggested a significant father-son PA relationship, similarly to the SOPHYA study and other studies [27,30,31]. The present study also confirms this significant association between mothers’ and daughters’ weekly PA. Although daughters’ PA is usually more influenced by mothers’ PA, a novel program to increase the PA of fathers and their daughters (DADEE) was tested. The results from the DADEE intervention program suggest that the appropriate engagement of fathers can increase PA in preadolescent girls [55]. Overall, we found that the mother-child relationship in PA decreased with the children’s age, but the father-child (in particular sons) relationship in PA was relatively stable. Although this observation is not supported by, for instance, a Swiss study of children aged 11–16 years, there are some similarities in the results with regard to the stability of the father-son PA relationship in those aged ten or more [30]. Generally, it can be expected that the association of PA of younger children who do not attend school yet with the PA of their parents could be stronger compared with that of older children, as they are not affected by the structure of the school day and because they are dependent on their parents, including engagement in PA with their parents. However, in the Czech Republic, preschool education is mostly state-run and free of charge for children starting at the age of three, and although attendance is not compulsory, it is widespread. Up to 88% of four-year-old Czech children attend a kindergarten [56]. All the preschool children in this study attended a kindergarten and therefore it cannot be assumed that they would spend more time with their parents during the school week than, for example, children in the first to the third grade, i.e., the differences in relationships could not arise as a result of more time spent with parents on weekdays.

The current study also analyzed parent-child SC relationships at weekends, on weekdays, and during the whole week. Generally, a stronger SC association was observed at weekends compared with working days, which is consistent with other studies that objectively measured PA in children and parents [35,57,58]. On the other hand, a Greek study of five-to-eight-year-old children involving 59 parent-child dyads reported a stronger parent-daughter PA relationship on weekdays compared with weekends [47]. However, a stronger parent-child relationship generally prevails at weekends, rather than on working days [35,57,58,59,60].

Converted to SC, weekly parent-child PA associations suggest that in children younger than eight years, an increase in the number of parents’ steps by 1000 per day may lead to an increase in the number of children’s steps by more than 400 steps per day. In the case of the older children in our study (8–16 years), we found a comparable effect in our study especially in the father-son dyads, which was also confirmed by similar results in parent-son dyads in the Canadian Physical Activity Levels among Youth (CANPLAY) study [61]. The results correspond with a study of ten-to-12-year-old children in which an increase in PA by one minute per day was associated with an increase in children’s PA by 0.21–0.24 min per day [30].

The strengths of this study are the seven-day continuous PA monitoring by objective tools in both children and their parents in a large sample and the addressing of the issue of parent-child associations in various children’s age categories and also by gender and the weekday-weekend variation. One of the limitations of the study is that, in line with the authors’ previous experiences, consent to involvement in the study was given primarily by relatively more physically active families; therefore we are hesitant to generalize the findings to families with generally low levels of PA. Another limitation is the possible reactivity effect in these types of studies when unsealed pedometers are used [39]. However, previous research shows that six days of unsealed pedometer data were found to be reliable for assessing habitual physical activity [39], and in monitoring lasting for at least four days no reactivity occurred [39,62,63]. In addition, to minimize the bias of the start day [43], the data from the first day of PA monitoring was not included in the analyses. Next, the long period of the data collection, during which the lifestyle of young people can change [64] could be understood as a limitation as well as the fact that the variables related to the socio-economic status of the families were not included in the current study. Last, the authors had limited information about parent-child coactivity, which could have provided further hints on the structure of mutual parent-child PA associations.

## 5. Conclusions

To conclude, parents play an important role in promoting their children’s active lifestyles. Although compared with other studies [30,33,54], the results are not always consistent, the present study suggested a strong relationship between the SC of parents and SC of children younger than eight years, the strongest being the relationship between mothers and daughters aged 4–7.9 years, especially at weekends. At a later age, the parent-child association is more affected by gender-based differences, with a dominant association especially between fathers and sons. An increase in the number of fathers’ steps by 1000 per day may lead to an increase in the number of their sons’ steps by about 400 per day. The parent-child associations were greater at weekends compared with weekdays. In summary, an increase in fathers’ and mothers’ physical activity, especially at weekends, may lead to an increase in the number of steps taken by their sons and daughters, respectively. Intervention programs aimed at the promotion of PA in children and adolescents should also involve both parents to increase the chance of being successful.

## Figures and Tables

**Table 1:** Basic characteristics and final numbers of parent-child dyads by age category and gender.

Children Age Category		Daughters	Sons	Mothers	Fathers
		M (SD)	M (SD)	M (SD)	M (SD)
**4–7.9 years**	**Calendar age (years)**	6.47 (1.07)	6.40 (0.99)	37.53 (4.37)	40.06 (4.93)
	**BMI (kg/m^2^)**	15.62 (2.56)	15.67 (1.99)	23.69 (3.77)	26.38 (3.56)
	**Mother-child dyads**	*n* = 161	*n* = 158		
	**Father-child dyads**	*n* = 103	*n* = 112		
**8–11.9 years**	**Calendar age (years)**	10.03 (1.19)	9.93 (1.17)	39.36 (4.02)	42.28 (5.13)
	**BMI (kg/m^2^)**	17.26 (2.73)	17.51 (2.92)	23.57 (3.72)	26.88 (3.30)
	**Mother-child dyads**	*n* = 238	*n* = 264		
	**Father-child dyads**	*n* = 151	*n* = 144		
**12–16 years**	**Calendar age (years)**	13.63 (1.06)	13.48 (1.04)	42.03 (4.72)	44.29 (5.91)
	**BMI (kg/m^2^)**	19.59 (2.94)	20.25 (3.53)	24.37 (3.78)	27.30 (3.72)
	**Mother-child dyads**	151	130		
	**Father-child dyads**	94	89		

Legend: BMI—Body mass index; M—Mean; SD—Standard deviation, *n*—number of participants in selected groups.

**Table 2:** Parent-child step count association (r_p_) by gender and age category.

r_p_		Mothers			Fathers		
Age Category		Week	Working Days	Weekend	Week	Working Days	Weekend
**4–7.9 years**	**Sons**	0.392 **	0.347 **	0.408 **	0.378 **	0.307 **	0.423 **
	**Daughters**	0.512 **	0.402 **	0.577 **	0.207 *	0.152	0.348 **
**8–11.9 years**	**Sons**	0.121	0.080	0.167 **	0.369 **	0.274 **	0.416 **
	**Daughters**	0.203 **	0.170 **	0.254 **	0.110	0.137	0.092
**12–16 years**	**Sons**	0.077	0.044	0.153	0.417 **	0.318 **	0.443 **
	**Daughters**	0.196 *	0.130	0.216 **	0.157	0.133	0.138

Legend: r_p_—Pearson correlation coefficient; * *p* < 0.05; ** *p* < 0.01.
